# Prediction of Treatment Response According to ASAS-EULAR Management Recommendations in 1 Year for Hip Involvement in Axial Spondyloarthritis Based on MRI and Clinical Indicators

**DOI:** 10.3389/fendo.2021.771997

**Published:** 2021-11-23

**Authors:** Zhuoyao Xie, Zixiao Lu, Hao Chen, Qiang Ye, Chang Guo, Kai Zheng, Xin Li, Qiuxia Xie, Shaoyong Hu, Quan Zhou, Yinghua Zhao

**Affiliations:** ^1^ Department of Radiology, The Third Affiliated Hospital of Southern Medical University (Academy of Orthopedics, Guangdong Province), Guangzhou, China; ^2^ Department of Computer Science and Engineering, The Hong Kong University of Science and Technology, Hong Kong, Hong Kong SAR, China

**Keywords:** magnetic resonance imaging, axial spondyloarthritis, hip involvement, treatment response, predictive model

## Abstract

**Background:**

To predict the treatment response for axial spondyloarthritis (axSpA) with hip involvement in 1 year based on MRI and clinical indicators.

**Methods:**

A total of 77 axSpA patients with hip involvement (60 males; median age, 25 years; interquartile, 22–31 years old) were treated with a drug recommended by the Assessment of SpondyloArthritis international Society and the European League Against Rheumatism (ASAS-EULAR) management. They were prospectively enrolled according to Assessment in SpondyloArthritis international Society (ASAS) criteria. Clinical indicators, including age, gender, disease duration, erythrocyte sedimentation rate (ESR) and C-reactive protein (CRP), were collected at baseline and in 3 months to 1-year follow-up. Treatment response was evaluated according to ASAS response criteria. MRI indicators consisting of bone marrow edema (BME) in acetabulum and femoral head, hip effusion, fat deposition, thickened synovium, bone erosion, bone proliferation, muscle involvement, enthesitis and bony ankylosis were assessed at baseline. Spearman’s correlation analysis was utilized for indicator selection. The selected clinical and MRI indicators were integrated with previous clinical knowledge to develop multivariable logistic regression models. Receiver operator characteristic curve and area under the curve (AUC) were used to assess the performance of the constructed models.

**Results:**

The model combining MR indicators comprising hip effusion, BME in acetabulum and femoral head and clinical indicators consisting of disease duration, ESR and CRP yielded AUC values of 0.811 and 0.753 for the training and validation cohorts, respectively.

**Conclusion:**

The model combining MRI and clinical indicators could predict treatment response for axSpA with hip involvement in 1 year.

## Introduction

Axial spondyloarthritis (axSpA) is a chronic autoimmune and autoinflammatory disease characterized by low back pain and morning back stiffness with the prevalence of 0.24% in Europe and 0.17% in Asia (1, 2). The severity of axial disease and poor long-term outcome are strongly correlated with hip involvement, which commonly accounts for 18% to 25% in axSpA patients (3). Generally, inflammation with bone marrow edema (BME), synovitis, bone erosions and osteophytes can affect hip joints, which would lead to ankylosis progression and functional impairment (3, 4).

The 2016 update of the Assessment of SpondyloArthritis international Society and the European League Against Rheumatism (ASAS-EULAR) firstly recommends non-pharmacological management and drug treatment for axSpA. Regarding drug treatment, nonsteroidal anti-inflammatory drugs (NSAIDs) and biological disease-modifying antirheumatic drugs (bDMARDs) are recommended for axSpA, whereas conventional synthetic disease-modifying antirheumatic drugs (csDMARDs) may be considered for axSpA patients with peripheral joints involvement (5, 6). However, not all axSpA patients with hip involvement are suitable for the recommendation because adverse events, including infections, gastrointestinal disorders and injection site reactions, might occur in some patients receiving drug treatment (2, 6). In particular, drug resistance in patients with systemic autoimmune disorders is still a challenge in treatment (7). Discontinuation or switching from drug therapy might lead to significantly worse clinical outcomes, including poor disease control and increased disease flares (8). Hence, predicting response to treatment and switching treatment plans before drug treatment help patients avoid adverse events and drug resistance.

Currently, hip involvement can be defined by different imaging techniques in axSpA patients, such as X-ray/computed tomography (CT) and magnetic resonance image (MRI) (4). Conventional radiography/CT can find structure damage and plays an essential role in the diagnosis and classification of axSpA, but it could not be used to detect early disease and predict treatment response (4). Comparatively, MRI could detect active inflammatory changes in early-stage axSpA, such as BME, synovitis, joint effusion and enthesitis (9). The Hip Inflammation MRI Scoring System (HIMRISS) is a feasible and reliable tool for evaluating BME and hip effusion (10). Some semi-quantitative MRI parameters of hip involvement proposed by radiologists have been used to evaluate disease progression and predict treatment response in axSpA (11, 12). Nonetheless, single MRI indicator could not predict treatment response in the individual axSpA patient with hip involvement. Berlin MRI spine score combined with symptom duration and C-reactive protein (CRP) could be used to predict response to drug treatment in axSpA (11, 12). However, an effective method for predicting therapy response for the hip involvement in axSpA to support physicians in clinical practice is still lacking.

We hypothesize that MRI indicators combined with clinical indicators might be associated with response to treatment for hip involvement in axSpA. Our study aimed to determine the potential predictive factors based on MRI and clinical indicators for treatment response in axSpA patients with hip involvement and build a robust model to predict whether the patient would benefit from pharmacological treatment in 1 year.

## Materials and Methods

### Patients

**Figure 1:**
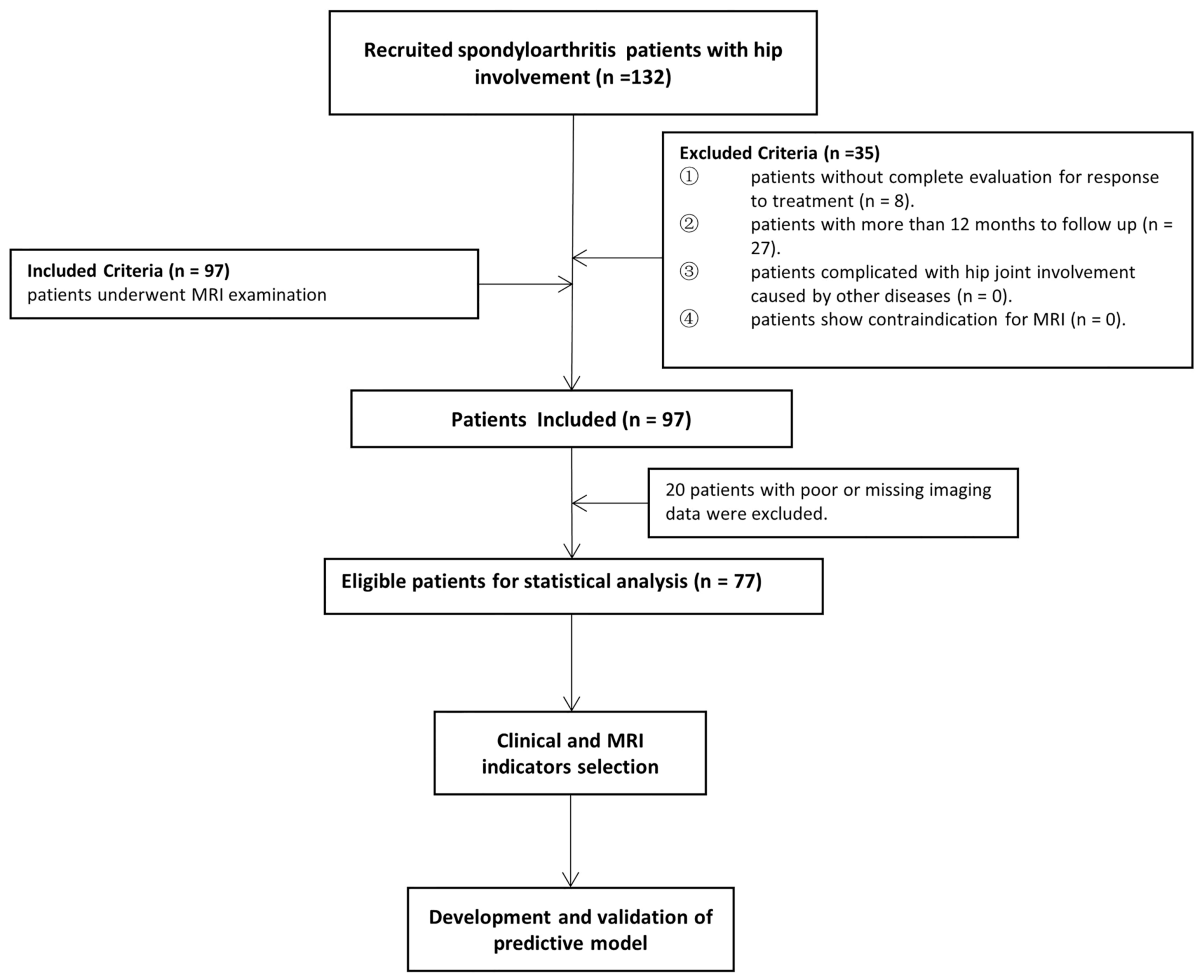
Flow chart of patients enrolled in the study.

According to the Assessment in SpondyloArthritis international Society (ASAS) (13), all consecutive axSpA patients with hip involvement treated with drugs at baseline were enrolled from January 2016 to June 2020 in our institution. An overview of the patient recruitment process is shown in **Figure 1**. Pharmacological treatment for patients were csDMARDs consisting of sulfasalazine and methotrexate (MTX) and bDMARDs, such as tumour necrosis factor inhibitor (TNFi) recommended by the ASAS-EULAR management (5). MR examination was performed within 2 weeks before or after treatment starting. Clinical indicators in axSpA patients at baseline and after 3 months to 1-year follow-up were obtained to evaluate treatment response. Hip involvement in our study was defined as abnormal MR findings in hip joint, such as bone marrow edema, effusion, fat deposition, thickened synovium, enthesitis, bone erosion, muscle involvement, bone proliferation and ankylosis. The patients were excluded in the following cases: (i) without more than two treatment response evaluation; (ii) lesions were located in other joints, such as shoulder, knee and ankle joints; (iii) hip involvement was accompanied with osteoarthritis, rheumatoid arthritis and other types of arthritis; (iv) poor or missing images for evaluation. Using 10-fold cross-validation, all patients were randomly divided into training and validation sets with a ratio of 9:1 according to treatment response.

### Clinical Indicator and Treatment Response

Clinical indicators included age, gender, disease duration, smoking status, history of drug treatment, presence of extra articular manifestations, human leukocyte antigen (HLA) B27, erythrocyte sedimentation rate (ESR), CRP, spinal pain, patient global assessment, Bath Ankylosing Spondylitis Disease Activity Index (BASDAI) and Bath Ankylosing Spondylitis Functional Index (BASFI). BASDAI and BASFI were described in **Appendix E1**, which were used to assess disease activity and function in axSpA, respectively (14, 15). Treatment response was assessed according to ASAS20, ASAS40, ASAS5/6 and ASAS partial remission improvement criteria (**Appendix E2**) (13).

### MRI Protocol

Clinical 3.0T system (Achieva 3.0T, Philips Healthcare, Best, Netherlands) was used to scan axSpA patients at baseline. Three axial MR sequences of the bilateral hip joints were conducted for the supine position. The sequence parameters are described in **Appendix E3**.

### Definition and Assessment of MRI Indicators

**Figure 2:**
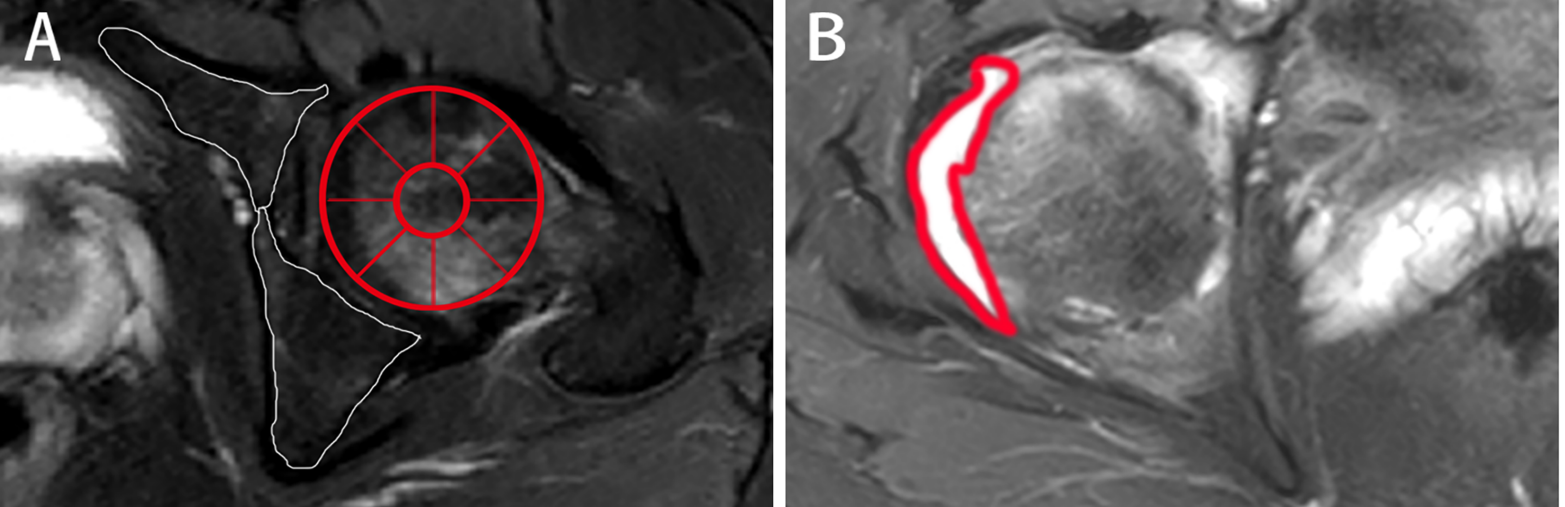
The scoring system on axial MRI in axSpA patients with hip involvement. **(A)** BME assessment in the two regions of left acetabulum delineated by white line and the nine regions of left femoral head segmented by red line is shown on SPAIR imaging, respectively. **(B)** Effusion assessment in the right hip is outlined by the thick red line on SPAIR imaging. AxSpA, Axial spondyloarthritis; BME, Bone marrow edema; SPAIR, Spectral attenuated inversion recovery.

The MRI indicators at hip joint were determined in axSpA patients with hip involvement, consisting of seven categorical, two semi-quantitative and one quantitative indicators. The methods for assessment of categorical MRI indicators, including fat deposition, thickened synovium, enthesitis, bone erosion, muscle involvement, bone proliferation and ankylosis are detailed in **Appendix E4**, and these indicators were recorded as present (1) or absent (0). The method to assess the semi-quantitative indicator BME at hip joint is described in **Appendix E5**. The quantitative indicator for hip joint, namely, hip effusion, is specified in **Appendix E6**. **Figure 2** demonstrates the scoring system for BME and hip effusion on axial MRI in hip joint. Inter-observer agreement for MR indicators in 30 patients was respectively assessed by two radiologists with 2 and 10 years of experience in musculoskeletal imaging (ZY.X and Q.Y). Both radiologists were blinded to the clinical indicators at baseline and the response to treatment within 1 year. Inter-observer agreement for MR indicators in 30 patients was independently evaluated twice by one radiologist (ZY.X). Controversial MRI assessments between the two radiologists were reviewed by a radiology expert (YH.Z, who has 30 years of experience in musculoskeletal imaging) to make an arbitration for further analysis. The remaining cases were independently evaluated by the radiologist (ZY.X) with 2 years of experience.

### Statistical Analysis

All data were analysed using Statistical Product and Service Solutions (SPSS) (version 22.0; IBM, Armonk, NY). Continuous variables of clinical data and MRI indicators were described as mean ± standard deviation or median (interquartile range [IQR]), and categorical variables were presented as binary numbers and n (%), as appropriate. The Cohen’s kappa coefficients and intraclass correlation coefficients (ICCs) were utilized to evaluate intra- and inter-observer agreement of categorical variables and continuous variables for MRI indicators, respectively. The Cohen’s kappa coefficients were descripted as follows: <0.20 (Poor), 0.21–0.40 (Fair), 0.41–0.60 (Moderate), 0.61- 0.80 (Good) and 0.81–1.00 (Perfect), respectively. Values less than 0.5, between 0.5 and 0.75 and greater than 0.75 indicated poor, moderate and good agreement for ICCs, respectively. The differences between the responders and non-responders were compared using the Mann-Whitney U test and Chi-square test with continuity correction, as appropriate. A two-tailed P value of less than 0.05 was regarded as statistically significant. MATLAB version 2018a was used to perform further analysis. To improve the performance of predictive models, we first normalized all the clinical and MRI indicator to a range of [-1,1] by subtracting the mean value of each indicator and dividing it by the standard deviation. Spearman’s correlation analysis was used to select indicators for further analysis. The multivariable logistic regression model was developed to find the best predictive linear combination of these indicators for predicting treatment response, thereby maximizing the conditional probability of the treatment response corresponding to the input data. All the models were trained and evaluated on the whole data set by using 10-fold cross-validation. Receiver operator characteristic (ROC) curve and area under the curve (AUC) were utilized to evaluate the models’ performance. The details regarding indicator selection and further developments and validation of the predictive model are described in **Appendixes E7** and **E8**, respectively.

## Results

### Patient Characteristics

A total of 132 patients were consecutively recruited from our institution in the study. A total of 55 patients were excluded because follow-up lasted for more than 12 months (n = 27), as well as insufficient images (n = 20) and incomplete clinical indicators to evaluate treatment response (n = 8). Seventy-seven patients (60 males and 17 females; median age, 25 years old; IQR, 22–31 years old) were ultimately enrolled for further analysis. The patient characteristics were summarized in **Table 1**. There were 4 (5.2%) patients without record of smoking status, history of drug treatment and extra articular manifestations and 6 (7.8%) patients without record of HLA B27 examination. 18 (23.4%) patients were treated with biologicals and 55 (71.2%) patients were not before our study, according to the ASAS-EULAR management before our study. All 77 patients obtained ASAS20 and ASAS40 results, whereas one patient lacked ASAS5/6 and ASAS partial remission results. The response rates evaluated according to ASAS20, ASAS40, ASAS5/6 and ASAS partial remission were 44.2%, 26.0%, 26.3% and 21.1%, respectively.

**Table 1:** Patient characteristics.

Clinical characteristics	Patients (n = 77)
Age (y)	25 (22-31)
Gender	
Male	60 (77.9%)
Female	17 (22.1%)
Disease duration (mon)	24 (11-60)
Smoking status	
Smoker	60 (1.3%)
Non-Smoker	72 (93.5%)
Extra-articular manifestation	
With	4 (5.2%)
Without	69 (89.6%)
History of drug treatments	
With	57 (74.0%)
Without	16 (20.8%)
HLA B27	
(+)	56 (72.7%)
(-)	15 (19.5%)
ESR (mm/h)	14 (6-32)
CRP (mg/L)	8 (3-21.5)

Continuous variables inconsistent with a normal distribution were presented as median (interquartile range). Categorical variables were presented as the number (percentage). HLA B27, human leukocyte antigen B27; ESR, erythrocyte sedimentation; CRP, C-reactive protein; BME, bone marrow edema.

### Clinical and MRI Indicators for Treatment Response Prediction

**Figure 3:**
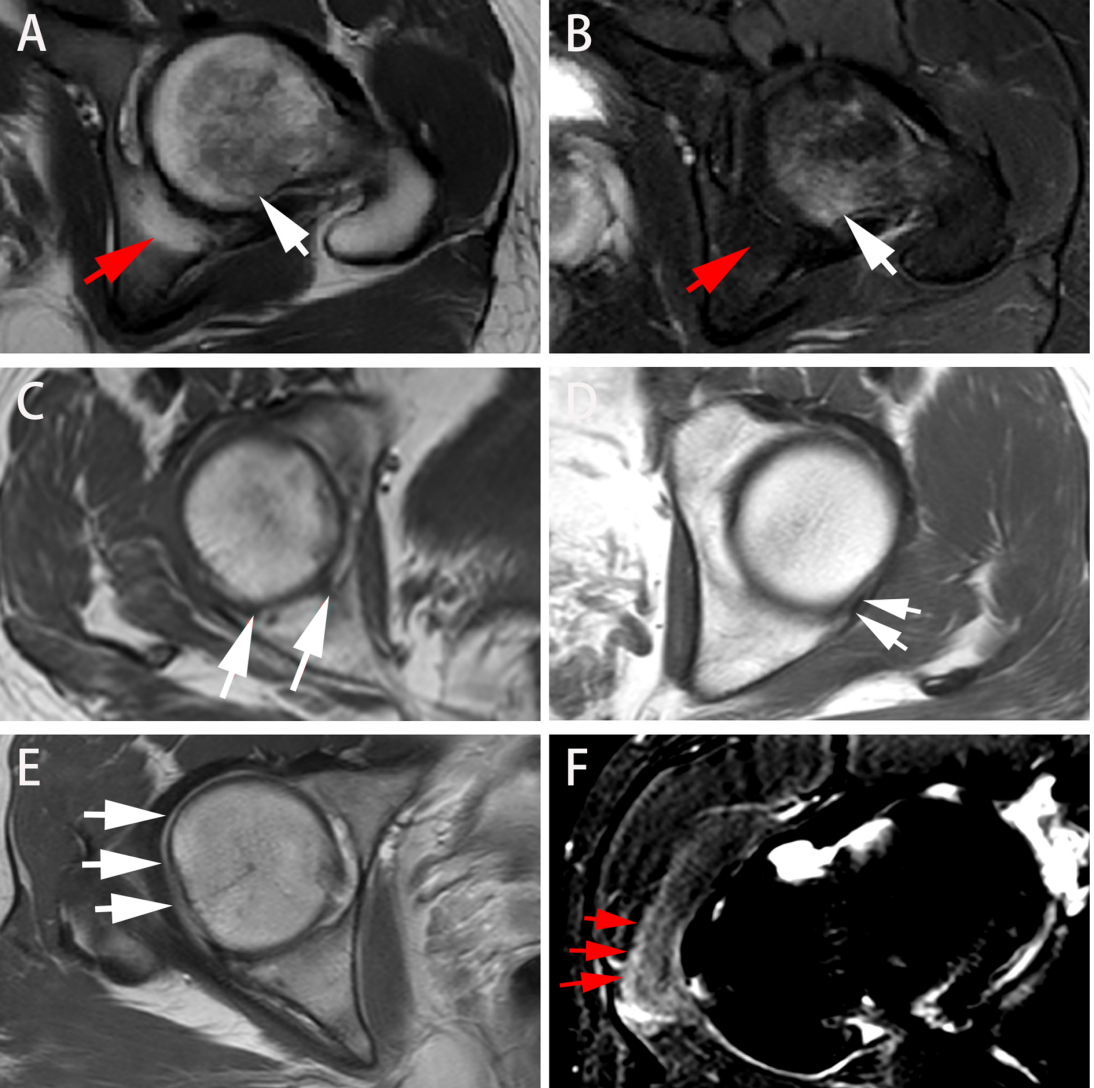
Different presentations on axial MRI in axSpA patients with hip involvement. **(A, B)** A 27-year-old male demonstrated BME (white arrow) in left femoral head and fat deposition (red arrow) in left acetabulum on T1WI and SPAIR imaging, respectively. **(C, D)** A 25-year-old male manifested erosion (arrows) in right acetabulum and bone proliferation (arrows) in left acetabulum, respectively. **(E)** A 16-year-old male revealed thickened synovium with slightly high signal strip in right hip on contrast-enhanced T1WI (arrows). **(F)** A 31-year-old male showed gluteus medius edema (arrows) in right hip on SPAIR imaging. AxSpA: Axial spondyloarthritis; BME: Bone marrow edema; T1WI: T1-weighted imaging; SPAIR: Spectral attenuated inversion recovery.

**Figure 3** displays various MRI findings in axSpA patients with hip involvement. Regarding MRI indicators in the 77 patients, hip effusion area, BME score in acetabulum and femoral head were 142 (94–210) mm**^2^**, 0 (0-5) and 0 (0–4), respectively. Forty-one (53.2%) patients presented fat a and 16 (20.8%) patients revealed thickened synovium. Bone proliferation appeared in 8 (10.4%) patients. Five (6.5%) patients displayed bone erosion. Five (6.5%) patients showed muscle involvement. In addition, enthesitis and bony ankylosis were not found. ICCs for the eight MRI indicators are tabulated in **Table 2**, and good observer agreements (ranged from 0.768 to 0.969) in hip joint evaluation were showed. Clinical and MRI indicators in responders and non-responders are summarized in **Table 3**. Four clinical and MRI indicators compromising disease duration (P = 0.038), ESR (P = 0.011), BME in acetabulum (P = 0.028) and hip effusion (P = 0.012) showed significant differences between responders and non-responders according to ASAS20. ESR (P = 0.044) and hip effusion (P = 0.019) were significantly different between responders and non-responders evaluated by ASAS40 criteria.

**Table 2:** Intra and inter-observer agreement for MRI assessments of two radiologists.

Parameters	Intra-observeragreement	95% CI	Inter-observeragreement	95% CI
BME in acetabulum	0.892	0.757-1.000	0.839	0.682-0.996
BME in femoral head	0.899	0.772-1.000	0.848	0.699-0.997
Hip effusion (mm^2^)	0.950	0.889-0.941	0.950	0.890-0.942
Fat deposition	0.867	0.691-1.000	0.800	0.590-1.000
Bone erosion	0.783	0.373-1.000	0.783	0.373-1.000
Bone proliferation	0.839	0.531-1.000	0.839	0.531-1.000
Thickened synovium	0.793	0.523-1.000	0.889	0.680-1.000
Muscle involvement	0.870	0.621-1.000	0.870	0.621-1.000

Calculation of Cohen’s kappa was performed for categorical variables. Intraclass correlation coefficients were applied for continuous variables; CI, confidence interval; BME, bone marrow edema.

**Table 3:** Comparison of clinical and MRI indicators of treatment responders and non-responders.

Parameters	ASAS20		*P*	ASAS40		*P*	ASAS5/6		*P*	ASAS partial remission		*P*
	Responder	Non-responder		Responder	Non-responder		Responder	Non-responder		Responder	Non-responder	
Clinical indicators												
Age (y)	25.5 (21.75-30.25)	25 (22-31)	0.939	25 (21-33.25)	25 (22-30.5)	0.825	26 (21.5-34.5)	24.5 (22-30.75)	0.457	25 (20.75-34)	25 (22-30)	0.853
Gender			0.779			0.497			0.521			0.957
Male	27 (79.4%)	33 (76.7%)		14 (70.0%)	46 (80.7%)		14 (70.0%)	45 (80.4%)		13 (81.3%)	46 (76.7%)	
Female	7 (20.6%)	10 (23.3%)		6 (30.0%)	11 (19.3%)		6 (30.0%)	11 (19.6%)		3 (18.8%)	16 (23.3%)	
Disease duration (mon)	24 (6-42)	35 (12-72)	0.038	24 (6-54)	27 (12-67.5)	0.213	24 (8.25-87)	25 (10.5-57)	0.714	24 (4-54)	25 (12-60)	0.561
Smoking status			1.000			1.000			1.000			1.000
Smoker	0 (0%)	1 (2.3%)		0 (0%)	1 (1.8%)		0 (0%)	1 (1.8%)		0 (0%)	1 (1.7%)	
Non-Smoker	32 (94.1%)	40 (93.0%)		20 (100%)	52 (91.2%)		20 (100%)	51 (91.1%)		15 (93.8%)	56 (93.3%)	
Extra-articular manifestation			1.000			0.641			1.000			1.000
With	2 (5.9%)	2 (4.7%)		2 (10.0%)	2 (3.5%)		1 (5.0%)	3 (5.4%)		1 (6.3%)	3 (5.0%)	
Without	30 (88.2%)	39 (90.7%)		18 (90.0%)	51 (89.5%)		19 (95.0%)	49 (87.5%)		14 (87.5%)	54 (90.0%)	
History of drug treatments			0.257			0.479			1.000			0.907
With	23 (67.6%)	34 (79.1%)		14 (70.0%)	43 (75.4%)		4 (20.0 %)	40 (71.4%)		11 (68.8%)	45 (75.0%)	
Without	9 (26.5%)	7 (16.3%)		6 (30.0%)	10 (17.5%)		16 (80.0%)	12 (21.4%)		4 (25.0%)	12 (20.0%)	
HLA B27			0.395			0.750			0.779			1.000
(+)	23 (67.6%)	33 (76.7%)		14 (70.0%)	42 (73.7%)		14 (70.0%)	41 (73.2%)		12 (75.0%)	43 (71.7%)	
(-)	8 (23.5%)	7 (16.3%)		5 (25.0%)	10 (17.5%)		5 (25.0%)	10 (17.9%)		3 (18.8%)	12 (20.0%)	
ESR (mm/h)	19 (10-46.25)	11 (3-27)	0.011	17.5 (11.5-48.75)	12 (3.5-29.5)	0.044	17.5 (10-47)	13.5 (4.25-29.5)	0.182	12.5 (6.25-19.75)	15 (4.25-36)	0.628
CRP (mg/L)	11.5 (5.5-20.25)	5 (0.5-26)	0.122	12.5 (4.5-20.75)	6 (0.5-22.5)	0.195	9.5 (4.5-20.75)	6 (0.63-21.75)	0.331	6.5 (3.25-16.5)	8.5 (0.5-24.5)	0.773
MRI indicators												
BME in acetabulum	2 (0-8)	2 (0-8)	0.028	4.5 (0-9.25)	0 (0-2.5)	0.056	0 (0-5.75)	0 (0-5)	0.979	0 (0-4.75)	0 (0-5)	0.578
BME in femoral head	0 (0-4)	0 (0-4)	0.602	0 (0-3.75)	0 (0-4)	0.810	0 (0-3.75)	0 (0-4)	0.823	0 (0-2)	0 (0-8.5)	0.287
Hip effusion (mm^2^)	168.5 (99.5-256)	128 (85-166)	0.012	181.5 (105.25-302)	137 (86-179)	0.019	181.5 (105.25-266)	137 (90.75-181.5)	0.066	133.5 (95.25-207.25)	149 (87.75-211)	0.736
Fat deposition	19 (55.9%)	22 (51.2%)	0.680	9 (45.0%)	32 (56.1%)	0.390	13 (65.0%)	28 (50.0%)	0.248	8 (50.0%)	33 (55.0%)	0.721
Bone erosion	2 (5.9%)	3 (7.0%)	1.000	0 (0%)	5 (8.8 %)	0.400	0 (0%)	5 (8.9%)	0.391	0 (0%)	5 (8.3%)	0.531
Bone proliferation	3 (8.8%)	5 (11.6%)	0.981	1 (5.0%)	7 (12.3 %)	0.623	1 (5.0%)	7 (12.5%)	0.607	1 (6.3%)	7 (11.7%)	0.866
Thickened synovium	7 (20.6%)	9 (20.9%)	0.971	5 (25.0%)	11 (19.3%)	0.826	4 (20%)	12 (21.4%)	1.000	3 (18.8%)	13 (21.7%)	1.000
Muscle involvement	2 (5.9 %)	3 (7.0%)	1.000	0 (0%)	5 (8.8%)	0.400	0 (0%)	5 (8.9%)	0.391	1 (6.3%)	4 (6.7%)	1.000

Continuous variables inconsistent with a normal distribution were presented as median (interquartile range). Categorical variables were presented as the number (percentage). The differences in continuous variables were compared using the Mann-Whitney U test. The differences in categorical variables were compared using the Chi-square test with continuity correction as appropriate. HLA B27, human leukocyte antigen B27; ESR, erythrocyte sedimentation; CRP, C-reactive protein; BME, bone marrow edema.

### Indicator Selection and Development of Predictive Model

Two steps were used for indicator selection. First, the four clinical and MRI indicators demonstrating significant differences in ASAS20 response evaluation were further analysed by Spearman’s rank correlation test, the results of which are tabulated in **Table 4**. Second, MRI indicators together with the clinical indicators were selected in accordance to the significant correlation with ASAS20 to build the logistic regression model for predicting treatment response. We constructed a primary model comprising disease duration, ESR, CRP and BME in the acetabulum. Disease duration, CRP, ESR and BME were reported as predictors of treatment response (12, 16). Referring to prior clinical knowledge, we considered that disease duration, ESR, CRP, hip effusion and BME in acetabulum and femoral head could be used to develop a predictive model.

**Table 4:** Results of indicators significantly correlated with treatment response.

Parameters	ASAS20		ASAS40		ASAS5/6		ASAS partial remission		
	*r*	*P*	*r*	*P*	*r*	*P*	*r*		*P*
Disease duration (mon)	-0.238	0.037	-0.143	0.215	0.042	0.717	-0.056		0.631
ESR (mm/h)	0.293	0.010	0.231	0.043	0.154	0.184	-0.067		0.565
BME in acetabulum	0.251	0.027	0.220	0.055	-0.003	0.979	-0.064		0.581
Hip effusion (mm^2^)	0.288	0.011	0.269	0.018	0.213	0.065	-0.039		0.738

ESR, erythrocyte sedimentation rate; BME, Bone marrow edema. “r”, Spearman correlation coefficient.

### Performance of Predictive Model of Treatment Response

**Figure 4:**
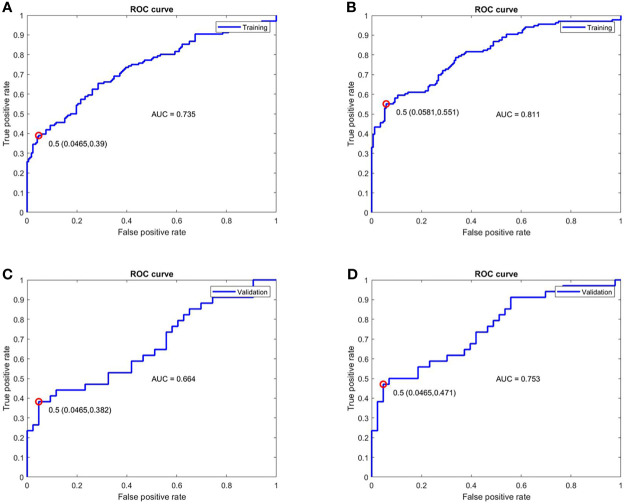
ROC analysis for the performance of the two models for treatment response prediction on the training and validation cohorts. **(A, C)** The model combining disease duration, ESR, hip effusion and BME in acetabulum showed AUC values of 0.735 and 0.664 for training and validation cohorts, respectively. **(B, D)** The model integrated disease duration, CRP, ESR, hip effusion and BME in the acetabulum and femoral head obtained better predictive ability with AUC values of 0.811 and 0.753 in the training and validation cohorts, respectively. ROC, Receiver operator characteristic; ESR, erythrocyte sedimentation rate; CRP, C-reactive protein; BME, Bone marrow edema; AUC, Area under the curve.

ROC analysis for the performance of predictive model is displayed in **Figure 4**. The model combining disease duration, ESR, CRP and BME in acetabulum yielded an AUC of 0.735 for the training set and an AUC of 0.664 for the validation set. The model integrating disease duration, ESR, CRP, hip effusion and BME in acetabulum and femoral head demonstrated AUC values of 0.811 and 0.753 for the training and validation sets, respectively.

## Discussion

In this prospective study, we developed and validated a model to predict curative effect before drug treatment in axSpA patients with hip involvement in 1 year. The model consisting of MRI (BME and hip effusion) and clinical indicators (disease duration, ESR and CRP) demonstrated optimal performance in predicting treatment response.

Our study firstly demonstrated that hip effusion (r = 0.288, P < 0.05) was the MR indicator with the highest number of correlations with responses to drug treatment in axSpA patients with hip involvement. No research has previously been conducted on the association between hip effusion and treatment response for axSpA. Gaffney et al.’s study validated that joint effusion could be used to predict pain response in patients with knee osteoarthritis (OA) treated with intra-articular steroid injections (IASI) at 1 and 6 weeks, respectively (17). Similarly, another study on knee OA stated that joint effusion was associated with the increased benefit of IASI treatment (18). Some researchers found that joint effusion-synovitis could not offer sufficient insight into clinical responses of patients with hip OA who were treated after 8 weeks and of RA patients treated after 2 years (19, 20). Although these studies involved various diseases and different joints, and the patients were treated with different drugs, the joint effusion was still an essential predictor of treatment response. The explicit association between hip effusion and the improvement in axSpA patients with hip involvement after drug treatment needs to be confirmed by others studies.

BME in acetabulum could be a predictor of good treatment response within 1 year. Additionally, BME in femoral head helped improve the performance of the predictive model, which indicated that it was a potential factor for response prediction. Similarly, Rudwaleit et al.’s finding indicated that BME evaluated by Berlin MRI spine score in axial joints contributed to the prediction of treatment response in axSpA patients (11, 12). A small sample size study concentrated on short-term predictive outcome of BME for radiographic progression from 6 to 12 months in rheumatoid arthritis (RA) (21). Hetland et al. identified BME as a predictor of progression at long-term follow-up in RA (20, 22). The predictive value of BME was not reported in the same rheumatic disease or in the follow-up interval in these studies, making direct comparisons with our study results difficult. Nevertheless, BME could still provide sufficient predictive value for patients before drug treatment. Notably, widespread active inflammation in the joint was manifested as BME on MRI in axSpA, OA and RA (23, 24); it was considered as an additional predictive value for treatment response in our study. Local inflammation in the involved joint (BME) could be visualized and assessed on MRI, whereas the inflammatory markers (CRP and ESR) could reflect the global inflammation disease, thereby implying that these predictors were complementary components. A previous study supported our results that a combination of BME and CRP demonstrated more powerful predictive capacity than a single parameter (12).

Fat deposition and bone erosion showed poor predictive value for treatment response in 1 year. However, Koo et al. demonstrated that fat deposition in the sacroiliac joint indicated a chronic stage of SpA, and the quantitative biomarker fat fraction was related to a worse outcome (25). No research has concentrated on the association between bone erosion and treatment response in SpA patients. However, similar to our results, research on RA patients indicated that bone erosion on MRI at baseline was not an independent predictor for radiographic progression in patients with early RA after 2 years (20). The follow-up time was relatively short in our study; thus, we were aware that long-term outcomes and life status after pharmacological treatment might be different from short-term results in SpA patients with hip involvement. More studies on long-term results are necessary, although they might be challenging. As thinner slices (2 or 3 mm) demonstrated high diagnostic accuracy and specificity for assessment of bone erosion on T1WI (26), 3 mm T1WI was applied to obtain a reliable and accurate assessment of bone erosion and to help identify the predictive value for treatment response in our study.

Higher ESR level and shorter disease duration predicted better treatment response. Despite that CRP showed no significance for prediction, it was reasonably added into the predictive model because of its advantage in response prediction (27), and the model performance was practically improved. These results were consistent with those of Lubrano et al., in which high ESR and CRP levels and low disease duration at baseline were confirmed to be significantly associated with improvement of BASFI (16), one of the four domains in the ASAS20 response criteria (13). Therefore, ESR and CRP levels could predict a better response to drug treatment in axSpA (28). Deodhar et al. confirmed our result that short disease duration was an important predictor for pharmacological response (29). ESR and CRP were important inflammatory markers but were limited because of their lack of sensitivity or specificity. Moreover, they might be affected by either non-infectious conditions or inflammation (30). Nonetheless, a combination of ESR and CRP with MRI indicators could improve the accuracy of the predictive model, which was currently used to help predict drug treatment response and guide patients’ treatment in clinical practice (27).

To our knowledge, this is the first time that MRI indicators were specifically considered in the construction of a predictive model for treatment response in axSpA patients with hip involvement. More evidence suggested that axSpA progression detected on MRI was related to treatment response (31). Inflammatory MRI findings on hip joint were an objective measurement whose association with inflammation on histopathology had been validated; they could serve as a reliable indicator (27). Some axSpA patients might benefit from MR examination before drug treatment; it could help prevent adverse events including infections and gastrointestinal disorder (2, 6), drug resistance (7), increased disease flares due to discontinuation of or switch from drug therapy (8) and the risk of undergoing total hip arthroplasty, i.e. heterotopic ossification and revision surgery (5, 32). We developed a predictive model consisting of six selected MRI and clinical indicators and showed that the model could successfully identify which patient can potentially benefit from a drug treatment with AUC values of 0.811 and 0.753 in training and validation cohorts, respectively. Thus, the constructed model could help rheumatologists discriminate responders and non-responders before treatment to increase the safety and effectiveness of drug therapy.

Several limitations should be acknowledged in our study. First, the sample size was relatively small in this single-centre study, which might lead to selection bias and influence the accuracy of our predictive model. Second, we used a scoring system similar to the HIMRISS and evaluated the hip joint on the axial plane instead of the coronal plane on MRI. Therefore, the reliability of our evaluation for hip joint and its correlation with the previous scoring system should be further tested. Third, many MRI indicators except for hip effusion were not quantitatively assessed, and assessment would be restricted by subjectivity and variability. Finally, the short-term effect (≤ 1 year) of treatment response was investigated in the study. As axSpA is a chronic condition, and continuous medication is regularly taken by patients (5), long-term outcomes of drug therapy need to be explored.

In conclusion, short disease duration, high ESR levels, hip effusion and BME in acetabulum predicted better treatment response within 1 year. The combination of MRI indicators, including hip effusion, BME in acetabulum and femoral head, and clinical indicators consisting of disease duration, ESR and CRP provided sufficient insights into response prediction. Prediction results would serve as promising guides in clinical decision-making for axSpA patients with hip involvement.

## Data Availability Statement

The raw data supporting the conclusions of this article will be made available by the authors, without undue reservation.

## Ethics Statement

The studies involving human participants were reviewed and approved by the institutional review board of the Third Affiliated Hospital of Southern Medical University (IRB number 201501003). Written informed consent from the participants’ legal guardian/next of kin was not required to participate in this study in accordance with the national legislation and the institutional requirements.

## Author Contributions

ZX: Conceptualization, methodology, validation, investigation, data curation, software, and writing – original draft. ZL: Methodology, software, validation, formal analysis, and writing – review and editing. HC: Validation. QY: Visualization. CG: Data curation and investigation. KZ Data curation. XL: Investigation. QX: Data curation. SH: Data curation. QZ: Resource. YZ: Conceptualization, methodology, visualization, validation, writing – review and editing. All authors contributed to the article and approved the submitted version.

## Funding

This study is supported by the National Natural Science Foundation of China (Grant No. 81871510).

## Conflict of Interest

The authors declare that the research was conducted in the absence of any commercial or financial relationships that could be construed as a potential conflict of interest.

## Publisher’s Note

All claims expressed in this article are solely those of the authors and do not necessarily represent those of their affiliated organizations, or those of the publisher, the editors and the reviewers. Any product that may be evaluated in this article, or claim that may be made by its manufacturer, is not guaranteed or endorsed by the publisher.

## References

[1] Sieper J Braun J Dougados M Baeten D. Axial Spondyloarthritis. Nat Rev Dis Primers (2015) 1:15013. doi: 10.1038/nrdp.2015.13 27188328

[2] Taurog JD Chhabra A Colbert RA. Ankylosing Spondylitis and Axial Spondyloarthritis. N Engl J Med (2016) 374(26):2563–74. doi: 10.1056/NEJMra1406182 27355535

[3] Wink F Arends S Maas F Bootsma H Griep EN Bruyn GAW. High Prevalence of Hip Involvement and Decrease in Inflammatory Ultrasound Lesions During Tumour Necrosis Factor-α Blocking Therapy in Ankylosing Spondylitis. Rheumatology (Ox Engl) (2019) 58(6):1040–6. doi: 10.1093/rheumatology/key382 30624693

[4] Vander Cruyssen B Vastesaeger N Collantes-Estévez E. Hip Disease in Ankylosing Spondylitis. Curr Opin Rheumatol (2013) 25(4):448–54. doi: 10.1097/BOR.0b013e3283620e04 23689637

[5] van der Heijde D Ramiro S Landewé R Baraliakos X Van den Bosch F Sepriano A. Update of the ASAS-EULAR Management Recommendations for Axial Spondyloarthritis. Ann Rheum Dis (2017) 76(6):978–91. doi: 10.1136/annrheumdis-2016-210770 28087505

[6] Navarro-Sarabia F Fernández-Sueiro JL Torre-Alonso JC Gratacos J Queiro R Gonzalez C. High-Dose Etanercept in Ankylosing Spondylitis: Results of a 12-Week Randomized, Double Blind, Controlled Multicentre Study (LOADET Study). Rheumatology (Ox Engl) (2011) 50(10):1828–37. doi: 10.1093/rheumatology/ker083 21700683

[7] Picchianti-Diamanti A Rosado MM Scarsella M Laganà B D'Amelio R. P-Glycoprotein and Drug Resistance in Systemic Autoimmune Diseases. Int J Mol Sci (2014) 15(3):4965–76. doi: 10.3390/ijms15034965 PMC3975434 24658440

[8] Wolf D Skup M Yang H Fang AP Kageleiry A Chao J. Clinical Outcomes Associated With Switching or Discontinuation From Anti-TNF Inhibitors for Nonmedical Reasons. Clin Ther (2017) 39(4):849–62.e6. doi: 10.1016/j.clinthera.2017.03.005 28363696

[9] Althoff CE Sieper J Song I-H Haibel H Weiß A Diekhoff T. Active Inflammation and Structural Change in Early Active Axial Spondyloarthritis as Detected by Whole-Body MRI. Ann Rheum Dis (2013) 72(6):967–73. doi: 10.1136/annrheumdis-2012-201545 22736088

[10] Maksymowych WP Cibere J Loeuille D Weber U Zubler V Roemer FW. Preliminary Validation of 2 Magnetic Resonance Image Scoring Systems for Osteoarthritis of the Hip According to the OMERACT Filter. J Rheumatol (2014) 41(2):370–8. doi: 10.3899/jrheum.131083 24241483

[11] van der Heijde D Landewé R Hermann K-G Rudwaleit M Østergaard M Oostveen A. Is There a Preferred Method for Scoring Activity of the Spine by Magnetic Resonance Imaging in Ankylosing Spondylitis? J Rheumatol (2007) 34(4):871–3. 17407242

[12] Rudwaleit M Schwarzlose S Hilgert ES Listing J Braun J Sieper J. MRI in Predicting a Major Clinical Response to Anti-Tumour Necrosis Factor Treatment in Ankylosing Spondylitis. Ann Rheum Dis (2008) 67(9):1276–81. doi: 10.1136/ard.2007.073098 18006539

[13] Sieper J Rudwaleit M Baraliakos X Brandt J Braun J Burgos-Vargas R. The Assessment of SpondyloArthritis International Society (ASAS) Handbook: A Guide to Assess Spondyloarthritis. Ann Rheum Dis (2009) 68 Suppl 2:ii1–44. doi: 10.1136/ard.2008.104018 19433414

[14] Garrett S Jenkinson T Kennedy LG Whitelock H Gaisford P Calin A. A New Approach to Defining Disease Status in Ankylosing Spondylitis: The Bath Ankylosing Spondylitis Disease Activity Index. J Rheumatol (1994) 21(12):2286–91. 7699630

[15] Calin A Jones SD Garrett SL Kennedy LG. Bath Ankylosing Spondylitis Functional Index. Br J Rheumatol (1995) 34(8):793–4. doi: 10.1093/rheumatology/34.8.793 7551671

[16] Lubrano E Perrotta FM Manara M D'Angelo S Ramonda R Punzi L. Improvement of Function and Its Determinants in a Group of Axial Spondyloarthritis Patients Treated With TNF Inhibitors: A Real-Life Study. Rheumatol Ther (2020) 7(2):301–10. doi: 10.1007/s40744-020-00197-5 PMC7211226 32062827

[17] Gaffney K Ledingham J Perry JD. Intra-Articular Triamcinolone Hexacetonide in Knee Osteoarthritis: Factors Influencing the Clinical Response. Ann Rheum Dis (1995) 54(5):379–81. doi: 10.1136/ard.54.5.379 PMC1005598 7794044

[18] Arden NK Reading IC Jordan KM Thomas L Platten H Hassan A. A Randomised Controlled Trial of Tidal Irrigation vs Corticosteroid Injection in Knee Osteoarthritis: The KIVIS Study. Osteoarthritis Cartilage (2008) 16(6):733–9. doi: 10.1016/j.joca.2007.10.011 18077189

[19] Steer KJD Bostick GP Woodhouse LJ Nguyen TT Schankath A Lambert RGW. Can Effusion-Synovitis Measured on Ultrasound or MRI Predict Response to Intra-Articular Steroid Injection in Hip Osteoarthritis? Skeletal Radiol (2019) 48(2):227–37. doi: 10.1007/s00256-018-3010-9 29980827

[20] Hetland ML Ejbjerg B Hørslev-Petersen K Jacobsen S Vestergaard A Jurik AG. MRI Bone Oedema Is the Strongest Predictor of Subsequent Radiographic Progression in Early Rheumatoid Arthritis. Results From a 2-Year Randomised Controlled Trial (CIMESTRA). Ann Rheum Dis (2009) 68(3):384–90. doi: 10.1136/ard.2008.088245 18388160

[21] Tamai M Arima K Nakashima Y Kita J Umeda M Fukui S. Baseline MRI Bone Erosion Predicts the Subsequent Radiographic Progression in Early Rheumatoid Arthritis Patients Who Achieved Sustained Good Clinical Response. Mod Rheumatol (2017) 27(6):961–6. doi: 10.1080/14397595.2017.1294280 28269999

[22] Hetland ML Østergaard M Stengaard-Pedersen K Junker P Ejbjerg B Jacobsen S. Anti-Cyclic Citrullinated Peptide Antibodies, 28-Joint Disease Activity Score, and Magnetic Resonance Imaging Bone Oedema at Baseline Predict 11 Years' Functional and Radiographic Outcome in Early Rheumatoid Arthritis. Scand J Rheumatol (2019) 48(1):1–8. doi: 10.1080/03009742.2018.1466362 30101636

[23] Manara M Varenna M. A Clinical Overview of Bone Marrow Edema. Reumatismo (2014) 66(2):184–96. doi: 10.4081/reumatismo.2014.790 25069499

[24] Narváez JA Narváez J de Albert M De Lama E Serrallonga M Nolla JM. Bone Marrow Edema in the Cervical Spine of Symptomatic Rheumatoid Arthritis Patients. Semin Arthritis Rheumatol (2009) 38(4):281–8. doi: 10.1016/j.semarthrit.2008.01.005 18336873

[25] Koo BS Song Y Shin JH Lee S Kim T-H. Evaluation of Disease Chronicity by Bone Marrow Fat Fraction Using Sacroiliac Joint Magnetic Resonance Imaging in Patients With Spondyloarthritis: A Retrospective Study. Int J Rheum Dis (2019) 22(4):734–41. doi: 10.1111/1756-185X.13485 30740910

[26] Chen M Herregods N Jaremko JL Carron P Elewaut D den Bosch FV. Diagnostic Performance for Erosion Detection in Sacroiliac Joints on MR T1-Weighted Images: Comparison Between Different Slice Thicknesses. Eur J Radiol (2020) 133:109352. doi: 10.1016/j.ejrad.2020.109352 33096409

[27] Maksymowych WP. Biomarkers for Diagnosis of Axial Spondyloarthritis, Disease Activity, Prognosis, and Prediction of Response to Therapy. Front Immunol (2019) 10:305. doi: 10.3389/fimmu.2019.00305 30899255 PMC6416369

[28] Arends S Brouwer E van der Veer E Groen H Leijsma MK Houtman PM. Baseline Predictors of Response and Discontinuation of Tumor Necrosis Factor-Alpha Blocking Therapy in Ankylosing Spondylitis: A Prospective Longitudinal Observational Cohort Study. Arthritis Res Ther (2011) 13(3):R94. doi: 10.1186/ar3369 21689401 PMC3218909

[29] Deodhar A Yu D. Switching Tumor Necrosis Factor Inhibitors in the Treatment of Axial Spondyloarthritis. Semin Arthritis Rheumatol (2017) 47(3):343–50. doi: 10.1016/j.semarthrit.2017.04.005 28551170

[30] Bray C Bell LN Liang H Haykal R Kaiksow F Mazza JJ. Erythrocyte Sedimentation Rate and C-Reactive Protein Measurements and Their Relevance in Clinical Medicine. WMJ (2016) 115(6):317–21. 29094869

[31] van der Heijde D. Radiographic Progression in Rheumatoid Arthritis: Does It Reflect Outcome? Does It Reflect Treatment? Ann Rheum Dis (2001) 60 Suppl 3:iii47–50. doi: 10.1136/ard.60.90003.iii47 PMC1766669 11890653

[32] Joshi AB Markovic L Hardinge K Murphy JCM. Total Hip Arthroplasty in Ankylosing Spondylitis: An Analysis of 181 Hips. J Arthroplasty (2002) 17(4):427–33. doi: 10.1054/arth.2002.32170 12066271

