# Supplementary material for: Prediction of Treatment Response According to ASAS-EULAR Management Recommendations in 1 Year for Hip Involvement in Axial Spondyloarthritis Based on MRI and Clinical Indicators
Source: Front Endocrinol (Lausanne). 2021 Nov 23;12:771997. doi: 10.3389/fendo.2021.771997 (PMC8650706; doi:10.3389/fendo.2021.771997)
Supplement: Supplementary file 1 [file DataSheet_1.docx]

Supplementary Material

**Supplementary Data**

**Appendix E1. BASDAI and BASFI**

| \| Please draw a mark on each line below to indicate your ability with each of the following activities, during the past week. \| \| --- \| \| 1. Putting on your socks or tights without help or aids (e.g. sock aids)?  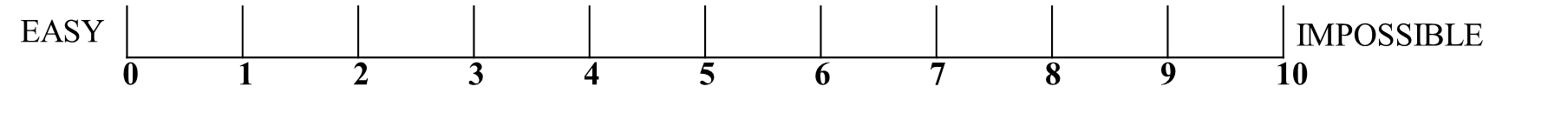  2. Bending forward from the waist to pick up a pen from the floor without an aid? \| \| 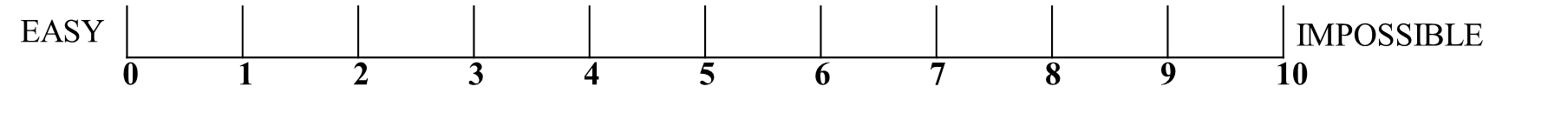3. Reaching up to a high shelf without help or aids (e.g. helping hand)? \| \| 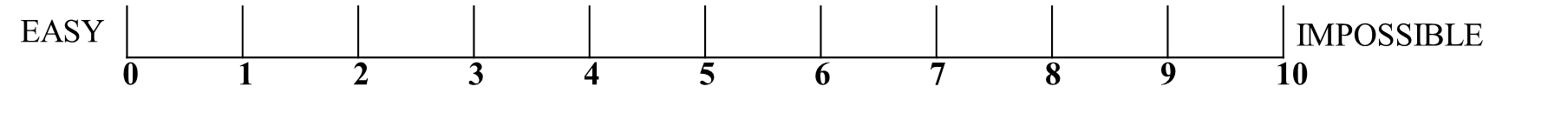 \| \| 4. Getting up out of an armless dining room chair without using your hands or any other help? \| \| 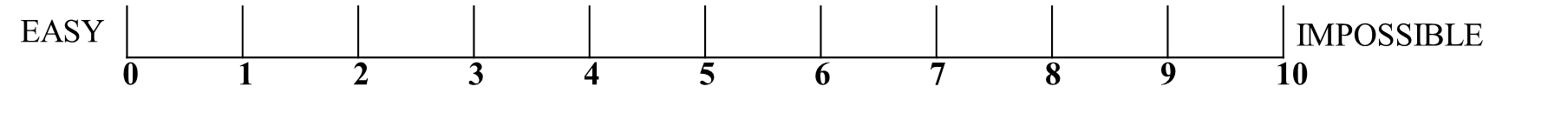 \| \| 5. Getting up off the floor without any help from lying on your back? \| \| 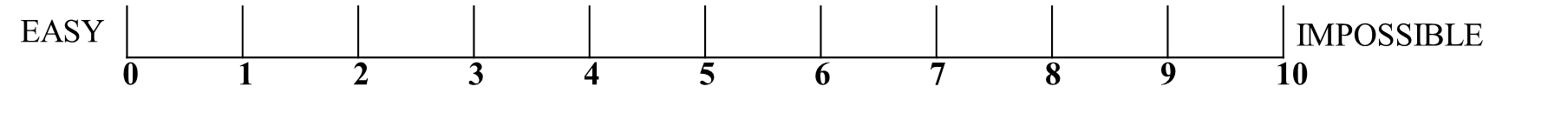 \| \| 6. Standing unsupported for 10 minutes without discomfort? \| \| 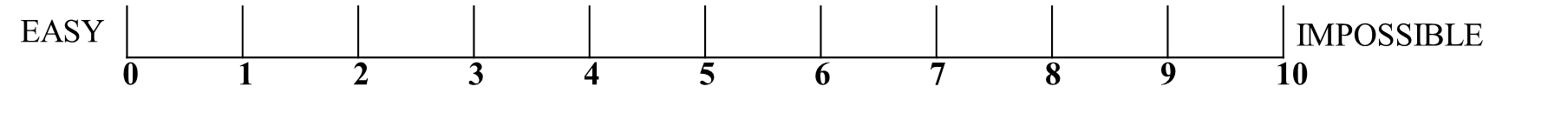 \| \| 7. Climbing 12-15 steps without using a handrail or walking aid (one foot on each step)? \| \| 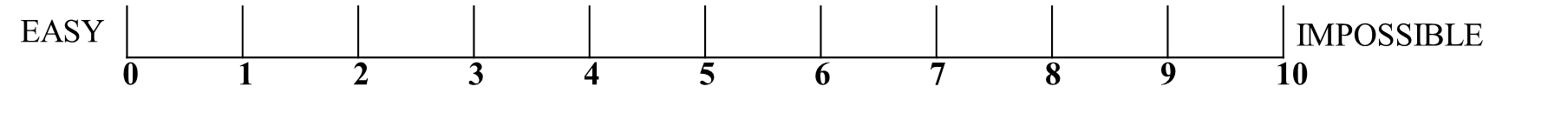 \| \| 8. Looking over your shoulder without turning your body? \| \| 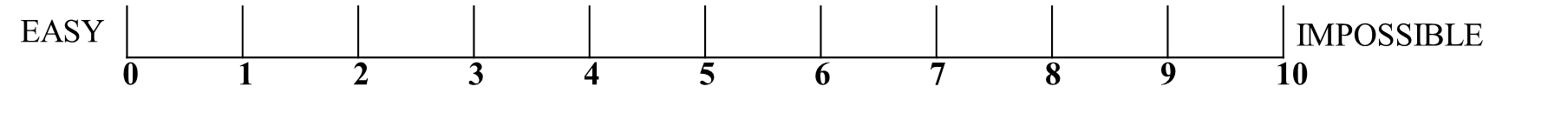 \| \| 9. Doing physically demanding activities (e.g. physiotherapy exercises, gardening or sports)? \| \| 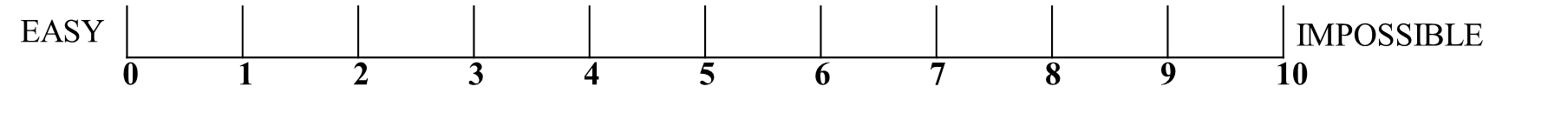 \| \| 10. Doing a full day activities whether it be at home or work? \| \| 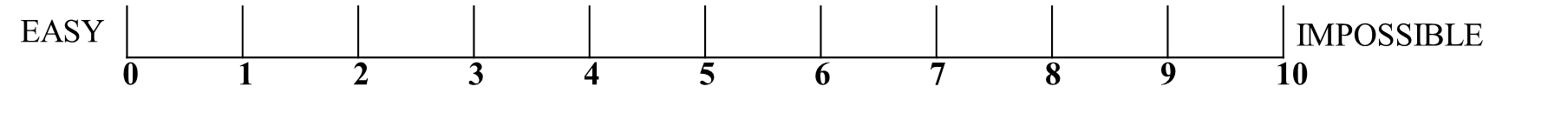 \| \| *Note:* The BASFI is the mean of 10 item scores completed on a numerical rating scale. \|   **The Bath Ankylosing Functional Index (BASFI)** |
| --- | --- | --- | --- | --- | --- | --- | --- | --- | --- | --- | --- | --- | --- | --- | --- | --- | --- | --- | --- |
| **The Bath Ankylosing Spondylitis Disease Activity Index (BASDAI)**  Please place a mark on each line below to indicate your answer to each question relating to the past week. |
| 1. How would you describe the overall level of fatigue/tiredness you have experienced?  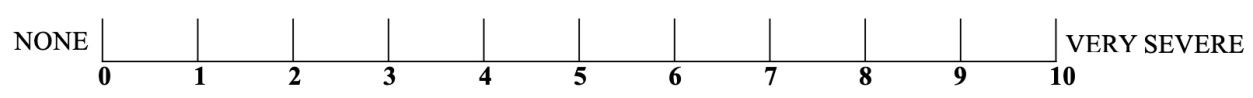 |
| 2. How would you describe the overall level of AS neck, back or hip pain you have had?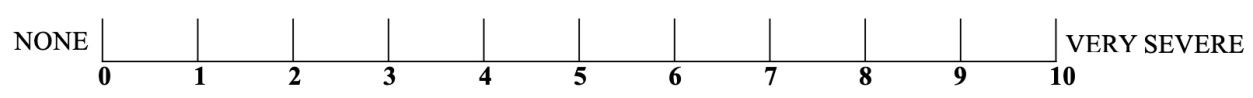 |
| 3. How would you describe the overall level of pain swelling in joints other than neck, back or hips you have had?  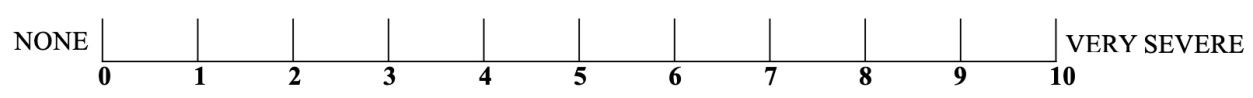 |
| 4. How would you describe the overall level of discomfort you have had from any areas tender to touch or pressure? |
| 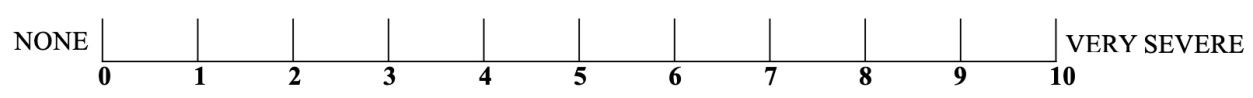 |
| 5. How would you describe the overall level of morning stiffness you have had from the time you wake up? |
| 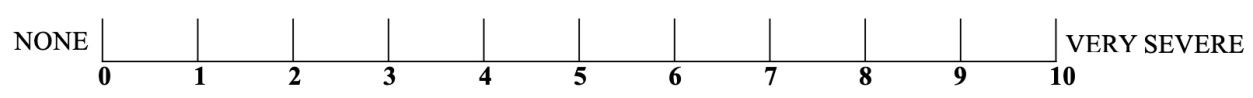  6. How long does your morning stiffness last from the time that you wake up? |
| NONE \| \| \| \| \| VERY SEVERE  0 0.5 1 1.5 2 or more hours |
| *Note:* Calculation of BASDAI: Calculate the sum of the values of question 1–4 and add the result to the mean of questions 5 and 6. Divide the result by 5. |

**Appendix E2.** **Assessment in SpondyloArthritis international Society 20% improvement (ASAS20), ASAS40, ASAS5/6 and ASAS partial remission response criteria** (1)

(i) ASAS20 improvement criteria:

Four domains: patient global, pain, function and inflammation.

Improvement of >20% and >1 unit in at least three domains on a scale of 10.

No worsening of >20% and >1 unit in the remaining domain on a scale of 10.

(ii) ASAS40 improvement criteria:

Four domains: patient global, pain, function and inflammation.

Improvement of >40% and >2 units in at least three domains on a scale of 10.

No worsening at all in the remaining domain.

(iii) ASAS partial remission criteria:

Four domains: patient global, pain, function and inflammation.

A value of no higher than 2 units in each of the domains on a scale of 10.

(iv) ASAS5/6 improvement criteria:

Six domains: patient global, pain, function, inflammation, CRP and spinal mobility.

Improvement of >20% in at least five domains.

**Appendix E3. MRI protocol**

The sequences were included as follows: (A) turbo spin echo T1-weighted imaging (TSE T1WI) (repetition time [TR]: 500 ms; echo time [TE]: 10 ms; section thickness: 3 mm; gap: 1 mm; field of view [FOV]: 300 × 400 mm^2^; matrix: 332 × 353); (B) T2-weighted imaging (T2WI) spectral attenuated inversion recovery imaging (SPAIR) (TR: 5200 ms; TE: 70 ms; section thickness: 5 mm; gap: 0.5 mm; FOV: 240 × 390 mm^2^; matrix: 284 × 435); (C) contrasted-enhanced (CE) T1WI (TR: 671 ms; TE: 20 ms; section thickness: 3 mm; gap: 1 mm; FOV: 200 × 360 mm^2^; matrix: 272 × 345) application of the intravenous injection of gadolinium agent at a dose of 0.2 mmol/kg of gadodiamide (Jiangsu Hengrui Medicine Co., Ltd., Jiangsu Province, China).

**Appendix E4. Assessment of categorical indicators for treatment response prediction**

Fat deposition was defined as high-intensity on T1WI located periarticularly. Enthesitis was hyperintense on T2WI SPAIR images and/or on CE T1WI at ligament and tendon attachment sites. Bone erosion was considered as defect and damage at the bone margin. Ankylosis occurred when adjacent bones directly fused with each other across the joint (2). Bone proliferation was irregular bone excrescence at the joint margin and subchondral sclerosis. Thickened synovium was presented as hyperintense after contrast medium administration in the hip joint space. Muscle involvement revealed a hyperintense signal on T2WI SPAIR images.

**Appendix E5. Assessment of semi-quantitative indicators for treatment response prediction**

BME was defined as an area of hypointensity or isointensity on T1WI and hyperintensity on T2WI, according to T2-weighted fat-suppressed imaging and CE T1WI within the bone marrow in the acetabulum and femoral head (3).

(i) BME score for femoral head: As for femoral head, the two central slices of the femoral head and the respective two slices that are superior and inferior to these central slices are counted on the horizontal plane. The two images with the longest diameter of the femoral head on the horizontal plane are regarded as the two most central slices. In the two central slices, the femoral head is divided into eight congruent regions of 45° angle with the middle as the 9th region. In addition, the femoral heads are halved into anterior and posterior regions for the two superior and the two inferior slices.

(ii) BME score for acetabulum: The acetabulum is segmented into two regions, namely, anterior and posterior, in the continuous six slice matching with the six slices of the femoral.

BME appearing in each region per slice is recorded as 1 score, and the total BME scores are 52 and 24 for the femoral head and acetabulum in bilateral hip joints, respectively.

**Appendix E6. Assessment of quantitative indicators for treatment response prediction**

Hip joint effusion was evaluated by applying the system supported by Mitchell et al. (4). Adequate fluid surrounding the femoral neck or swelling joint capsules in hip joints on STIR and T2-weighted images was regarded as joint effusion. The total area of the ROIs in the same slice was aggregated to obtain the effusion area of the slice. The maximal area in one slice was selected as the representative effusion area of the hip (5).

**Appendix E7. Clinical and MRI indicators selection**

To determine the univariate indicators mostly related to treatment response, Spearman's correlation analysis was used to explore the associations between the 13 clinical and MRI indicators and treatment responses. A *P* value smaller than 0.05 was considered statistically significant. Then, the clinical and MRI indicators demonstrating significant correlation with treatment response were selected for the multivariate analysis.

**Appendix E8. Development and validation of predictive model**

(i) We aimed to find a linear combination of clinical and MRI indicators in a multivariable model to maximise the conditional probability of the treatment response status {0,1} corresponding to the input data. The logistic regression was used as the multivariable model in our study. Based on the results of the previous univariate analysis, the selected four clinical and MRI indicators were used to establish four independent logistic regression models of the order 1–4, where the order of each model represents the number of indicators to be selected as variables. Our logistic regression model can be defined as follows:

$$g\left( X_{i} \right)=\beta_{0}+\sum_{j=1}^{b} \beta_{j}x_{ij}, i=1, 2, \cdots N$$

where $x_{ij}$ represents the $j$th indicator of the $i$th patient selected from the univariate analysis. $N$ represents the number of patients in the training set. $b$ represents the order of the model with a value ranging from 1 to 4. $\beta=\{\beta_{j}\in R:j=0, 1, \cdots b\}$ represents the coefficient set of the regression model.

Each order of model was trained and evaluated on the entire data set in 10-fold cross-validation, and the AUC values of the training and evaluation sets were calculated by ROC analysis. The model with the largest AUC value in the evaluation set was selected as the final multivariate model and was denoted as $M_{multi}$. The corresponding dependent variable set in $M_{multi}$ was recorded as $X_{multi}$. ROC analysis was based on the true positive rate (${TP}_{rate}$) and false positive rate (${FP}_{rate}$), which were defined as follows:

$${TP}_{rate}=\frac{TP}{TP+FN}, {FP}_{rate}=\frac{FP}{TN+FP}$$

where TP represents the number of positive samples that were correctly classified. False negative (FN) represents the number of negative samples that were incorrectly classified. FP represents the number of positive samples that were incorrectly classified. True negative (TN) represents the number of negative samples that were correctly classified. In this experiment, patients with treatment response status of 1 and 0 were regarded as positive and negative samples, respectively.

(ii) To improve the accuracy for predicting treatment response status, we further constructed a mixed multivariate model by introducing prior clinical knowledge in the indicator selection step. Previous literature (6, 7) has proven that five clinical and MRI indicators, namely, disease duration, CRP, ESR, BME in acetabulum and femoral head, were related to the treatment response status. Denoting these five clinical and MRI indicators as $X_{clinic}$, we first used $X_{clinic}$ to build a multivariate logistic regression model with an order of 5, denoted as $M_{clinic}$. Still, we performed the 10-fold cross-validation to train and evaluate this model on the whole dataset.

Next, indicators in $X_{multi}$ derived from the multivariate analysis were sorted by their corresponding regression coefficients in $M_{multi}$. The indicators overlapping with $X_{clinic}$ were eliminated from $X_{multi}$, and the remaining indicator set was marked as $X_{multi\_best}$. Similarly, to obtain the optimal indicator combination, different numbers of indicators from $X_{multi\_best}$ were successively selected and integrated with $X_{clinic}$ to establish logistic regression models of different orders and compare them with one another. Here, our mixed multivariable model can be defined as follows:

$$g\left( X_{i}^{'} \right)=\beta_{0}^{’}+\sum_{k=1}^{5} \beta_{k}^{'}x_{ik}^{'}+\sum_{j=0}^{b^{'}} \beta_{j}^{'}x_{ij}^{'}, i=1, 2, \cdots N$$

where $x_{ik}^{'}$ denotes the $k$ th indicator in $X_{clinic}$ of the $i$ th patient, $x_{ij}^{'}$ denotes the $j$ th indicator in $X_{multi\_best}$ of the $i$ th patient.$b^{‘}$ denotes the number of indicators in $X_{multi\_best}$ selected as variables of the current model and the value of $b^{‘}$ ranges from 0 to the total number of indicators contained in $X_{multi\_best}$. $\beta^{‘}=\{\beta_{k}^{'}\in R:k=0, 1, \cdots5, \beta_{j}^{'}\in R:j=0, \cdots b^{'} \}$ represents the regression coefficient set of the model. We performed ROC analysis on each model of different orders and selected the model with the largest AUC as the final optimal model.

**References**

1. Sieper J, Rudwaleit M, Baraliakos X, Brandt J, Braun J, Burgos-Vargas R, et al. The Assessment of SpondyloArthritis international Society (ASAS) handbook: a guide to assess spondyloarthritis. *Ann Rheum Dis*. (2009) 68 Suppl 2:ii1-i44. doi: 10.1136/ard.2008.104018.

2. Huang ZG, Zhang XZ, Hong W, Wang GC, Zhou HQ, Lu X, et al. The application of MR imaging in the detection of hip involvement in patients with ankylosing spondylitis. *Eur J Radiol*. (2013) Sep82(9):1487-93. Epub 2013/05/18. doi: 10.1016/j.ejrad.2013.03.020.

3. Patel S. Primary bone marrow oedema syndromes. *Rheumatology (Oxford, England)*. (2014) 53(5):785-792. doi: 10.1093/rheumatology/ket324.

4. Mitchell DG, Rao V, Dalinka M, Spritzer CE, Gefter WB, Axel L, et al. MRI of joint fluid in the normal and ischemic hip. *AJR Am J Roentgenol*. (1986) 146(6):1215-1218. doi: 10.2214/ajr.146.6.1215.

5. Wang X, Blizzard L, Jin X, Chen Z, Zhu Z, Han W, et al. Quantitative Assessment of Knee Effusion-Synovitis in Older Adults: Association With Knee Structural Abnormalities. *Arthritis Rheumatol*. (2016) 68(4):837-844. doi: 10.1002/art.39526.

6. Rudwaleit M, Schwarzlose S, Hilgert ES, Listing J, Braun J, Sieper J. MRI in predicting a major clinical response to anti-tumour necrosis factor treatment in ankylosing spondylitis. *Ann Rheum Dis*. (2008) 67(9):1276-1281. doi: 10.1136/ard.2007.073098.

7. Lubrano E, Perrotta FM, Manara M, D'Angelo S, Ramonda R, Punzi L, et al. Improvement of Function and Its Determinants in a Group of Axial Spondyloarthritis Patients Treated with TNF Inhibitors: A Real-Life Study. *Rheumatol Ther*. (2020) 7(2):301-310. doi: 10.1007/s40744-020-00197-5.
